# A High Aggression Strategy for Smaller Males

**DOI:** 10.1371/journal.pone.0043121

**Published:** 2012-08-14

**Authors:** P. Andreas Svensson, Topi K. Lehtonen, Bob B. M. Wong

**Affiliations:** 1 Department of Natural Sciences, Linnaeus University, Kalmar, Sweden; 2 School of Biological Sciences, Monash University, Victoria, Australia; 3 Section of Ecology, Department of Biology, University of Turku, Turku, Finland; Institut Pluridisciplinaire Hubert Curien, France

## Abstract

Male-male conflict is common among animals, but questions remain as to when, how and by whom aggression should be initiated. Factors that affect agonistic strategies include residency, the value of the contested resource and the fighting ability of the two contestants. We quantified initiation of aggression in a fish, the desert goby, *Chlamydogobius eremius*, by exposing nest-holding males to a male intruder. The perceived value of the resource (the nest) was manipulated by exposing half of the residents to sexually receptive females for two days before the trial. Resident male aggression, however, was unaffected by perceived mating opportunities. It was also unaffected by the absolute and relative size of the intruder. Instead resident aggression was negatively related to resident male size. In particular, smaller residents attacked sooner and with greater intensity compared to larger residents. These results suggest that resident desert goby males used set, rather than conditional, strategies for initiating aggression. If intruders are more likely to flee than retaliate, small males may benefit from attacking intruders before these have had an opportunity to assess the resident and/or the resource.

## Introduction

Male aggression is widespread among animals and can have a direct bearing on male reproductive success. At the same time, aggression can be very taxing, and animals have evolved various strategies to avoid physical combat and its associated costs. Considerable research has been focused on understanding male strategies for the initiation and escalation of aggressive behaviours. A number of important factors have been identified that influence whether or not a particular individual should initiate aggression [1], [2]. These typically relate to asymmetries between the two contestants, such as asymmetry in resource holding potential (RHP), often determined by body size asymmetry in information about the 46 contested resource or opponent, and asymmetry in the value placed on the resource. For example, both previous fighting experience [3], information about the opponent [4], and value placed on the resource [5] can affect the probability of an individual initiating aggression.

The classic game-theoretical framework of Maynard Smith and Parker [1] suggests that conflicts should mainly be resolved through an ordered exchange of agonistic displays. Such displays are thought to facilitate mutual assessment of resource holding potential (RHP), ensuring that more costly types of aggression are avoided, unless the contestants are evenly matched. In practice, however, animals often need to decide on a course of action while still having imperfect information about their opponent or of the contested resource. Furthermore, it may not always be in the interest of a contestant to partake in mutual assessment. In particular, individuals with low RHP may want to avoid drawn-out rituals that can reveal their weakness to an opponent [6]. Similarly, when there is an asymmetry in residency (that is, when an resident confronts an intruder), it may be advantageous for the resident to attack before the intruder has had time to assess the resource, especially if the resident has low RHP. Confronted with an aggressive resident, the intruder may flee rather than stay and fight. For example, in the mangrove killifish, *Kryptolebias marmoratus*, a large proportion of attacked individuals will retreat immediately from the aggressor [3]. Likewise, territory-holding northern harriers, *Circus cyaneus*, are able to successfully drive off competing raptor species much larger than themselves [7].

Resource value is often seen as a “key component” of strategic models of aggression [8]. Defenders have been shown to increase their efforts as the value of the resource increases [9], [10]. Increasing the value of the resource does not simply increase aggression, but may also modify the strategy for aggression toward intruders [11]. Males defending a breeding resource are expected to be more aggressive if they have direct mating opportunities. For example, in butterflies, males that had recently encountered females were more motivated to defend their territory [12]. In autumn spiders, *Metellina segmentata*, residents fought longer over more fecund females, indicating that the perceived resource value affected fighting effort [13]. Similarly, in dung flies, *Scathophaga stercoraria*, intruders are more aggressive if the contested female is larger [9], and in skinks, *Eumeces laticeps*, males aggressively exclude other males from the vicinity of females [14].

Males of many species defend territories or breeding resources, such as nests. For example among substrate brooding fishes, where males also often provide exclusive care of the eggs, nesting substrates are often aggressively defended against other males. Such species are excellent models for testing predictions of male-male aggression when there are asymmetries associated with residency. The aggression strategy of a resident (nest holding) male toward an intruder can be affected by the availability of nest sites, body size asymmetry, and resource value. In addition, longer ownership increases aggression in nest defence [15]. Understanding this aggression is important, because fighting over nests can be very costly, and can significantly shorten the resident’s life span [16].

The Australian desert goby, *Chlamydogobius eremius*, is a freshwater fish endemic to the arid region of Central Australia [17]. As with most other gobies [18], desert gobies spend most of the day in shelters, such as crevices and under rocks. Males aggressively defend these daytime shelters, especially when they are also suitable as nest substrates. Indeed, nest holding males will typically attack other males, regardless of whether eggs are present in the nest. In the current study, we used the desert goby as a model to redress gaps in empirical knowledge on initiation of aggressive behaviour. Specifically, we investigated the strategies of nest holding males to initiate aggression when confronted with an intruding male, and tested whether an increase in perceived mating opportunities affects the level of aggression. We predicted that the presence of females (i.e. an increase in perceived mating opportunities) would increase the value of the nest to the resident and would cause an increased level of aggression. We also predicted that the size of the intruder (either absolute size or relative to the resident) would influence the aggression strategy of the resident.

**Figure 1:**
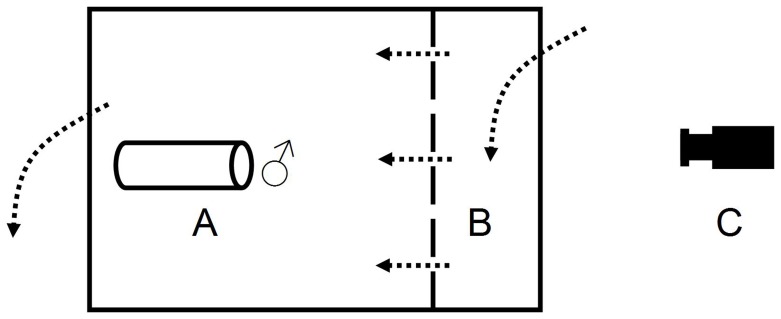
Top-view of the experimental setup. A) the resident male and the nest substrate. B) the compartment to which females and intruder males were introduced. C) the position of the video camera. The dashed line denotes the transparent divider and the dotted arrows demonstrate the direction of water flow through the tank.

## Materials and Methods

### Model System

The desert goby is a small (<8 cm), colourful species with paternal egg-care. It can be locally abundant in both permanent and temporary bodies of water, from spring-fed pools to ephemeral desert streams (Allen et al. 2002). Nest-holding males perform elaborate courtship displays to attract females to spawn [19], [20], [21], whereas rivals are expelled aggressively. Aggression typically occurs in distinctive “bouts”, which consist of the resident male darting out from the nest, repeatedly attacking the intruding male and then returning back to the nest. If males are allowed to interact physically, aggression can escalate to combat with males locking jaws and grappling with their opponent. In such fights, the winner will typically be the larger male (authors’ personal observations).

**Table 1:** Deleted terms and order or deletion from hierarchical simplification of three statistical models of male-male aggression.

Response	Time to first attack				Intensity of first attack bout				Number of attacks^a^			
Model type	Cox proportional hazardsregression model				Linear model				Linear model			
Variable	order	χ^2^	df	P	order	F	df	P	order	F	df	P
T:RMS:IMS	1	0.30	1	0.58	1	0.32	12,13	0.58	1	0.09	22,23	0.77
T:RMS	2	<0.01	1	0.96	4	<0.01	13,14	0.99	2	0.13	23,24	0.72
T:IMS	3	0.45	1	0.50	3	<0.01	14,15	0.96	3	1.23	24,25	0.28
RMS:IMS	4	0.60	1	0.44	2	0.18	15,16	0.68	4	0.93	25,26	0.34
IMS	5	0.03	1	0.85	5	0.40	16,17	0.53	5	0.14	26,27	0.71
T	6	0.56	1	0.46	6	2.11	17,18	0.16	6	1.20	27,28	0.28

The predictor variables were treatment (T, presence or absence of females prior to trial), resident male size (RMS), intruding male size (IMS) and all their interactions.

a The response variable was square-root transformed.

**Figure 2:**
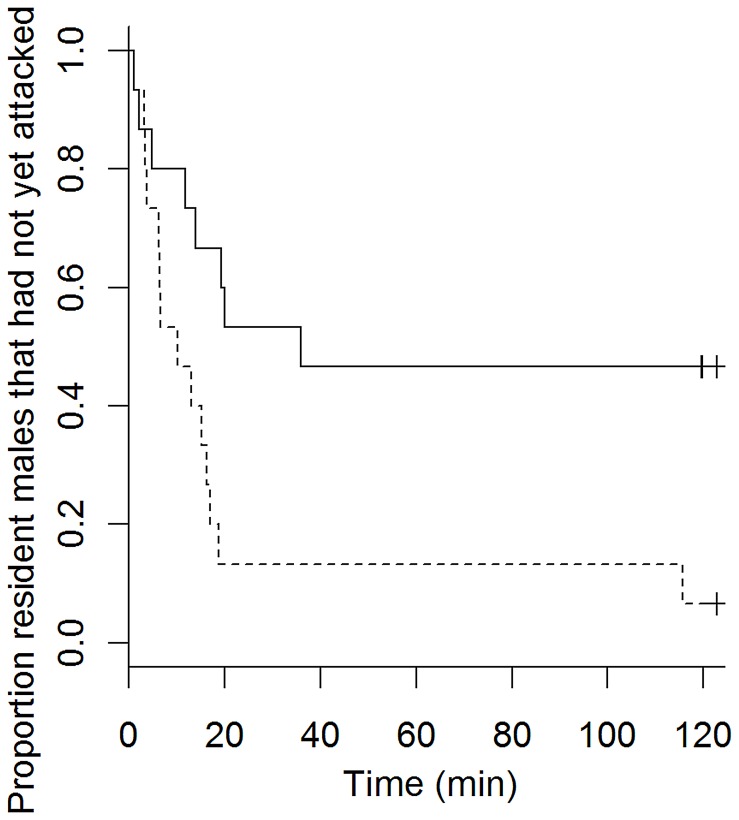
Kaplan-Meier survival curves for time to first attack after dividing resident males into large and small individuals. Solid line: resident males that were larger than the median length (TL = 62.2 mm). Dashed line: resident males smaller than median length. Crosses (+) indicate censored data.

**Table 2:** Probability of a non-zero coefficient of predictors (Pr_inc_) determined by Bayesian model averaging in three statistical models of male-male aggression.

Response	Time to first attack	Intensity of first attack bout		Number of attacks
Predictor		*all data two outliers removed*		
T	0.15	0.21	0.16	0.14
RMS	**0.82**	0.46	0. 71	**0.82**
IMS	0.25	0.19	0.25	0.27
T:RMS	0.15	0.22	0.17	0.14
T:IMS	0.16	0.22	0.16	0.16
RMS:IMS	0.29	0.33	0.33	0.32
T:RMS:IMS	0.16	0.22	0.16	0.16

The predictor variables were treatment (T, presence or absence of females prior to trial), resident male size (RMS), intruding male size (IMS) and all their interactions. Bold values indicate important predictors (Pr_inc_ >0.75).

### Fish Origin and Housing

Desert gobies, originating from waterholes and springs west of Lake Eyre in South Australia, were housed in single-sex holding tanks. All aquaria were kept at a temperature of 24–26°C on a 12 hour light: dark cycle and a salinity of 5‰ to mimic field conditions. Fish were fed daily on a diet of commercially prepared pellets and frozen *Artemia*. In the male holding tanks, rocks, plants and shelters (halved flower pots) were provided. The experiment was carried out in June and July of 2009.

### Experimental Design

Thirty males in breeding coloration were removed from holding tanks and placed in individual aquaria (length × width) 30×20 cm. These were filled to a depth of 13 cm with water and contained a nest in the form of a 9 cm long PVC pipe (3 cm diameter), secured by a ceramic tile that was buried into the gravel substrate [19], [20]. These tanks shared a re-circulating water supply with a central filter. The males were allowed to acclimatize to the new tanks for one week. A transparent divider was affixed 7 cm from the far end of the tank, dividing each tank into two compartments (Figure 1). In addition, an opaque plastic sheet was placed to cover the transparent divider. The flow of water went from the smaller compartment to an outlet behind the resident male’s nest. The transparent divider had holes to allow water flow and transmission of olfactory cues. The males (hereafter referred to as “resident males”) were haphazardly assigned to one of two treatments (*n* = 15 per treatment).

**Figure 3:**
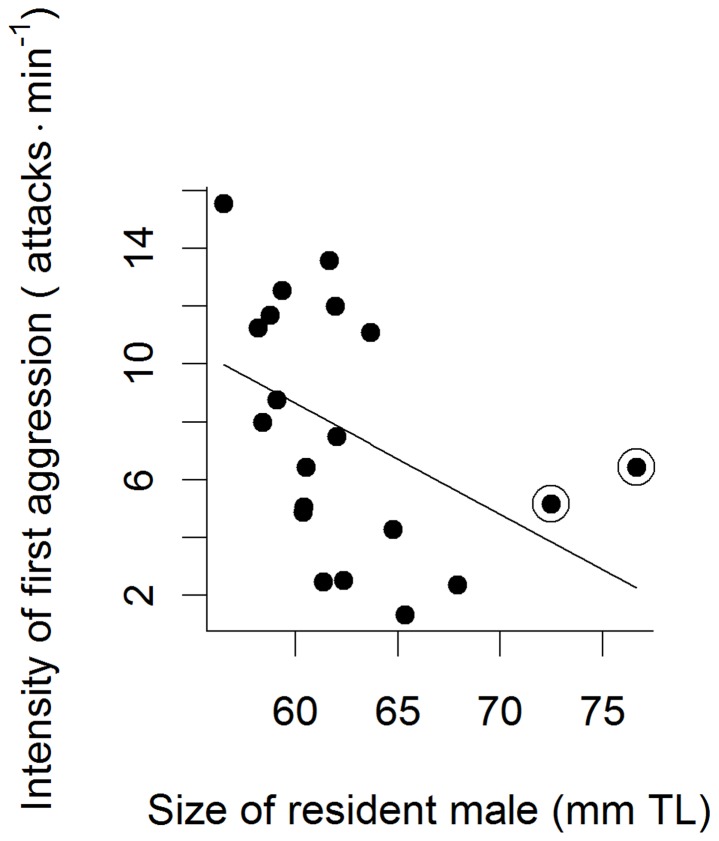
Relationship between the intensity of the first aggressive bout and resident male size in male Australian desert gobies (*Chlamydogobius eremius*). The circled symbols indicate two influential outliers.

**Figure 4:**
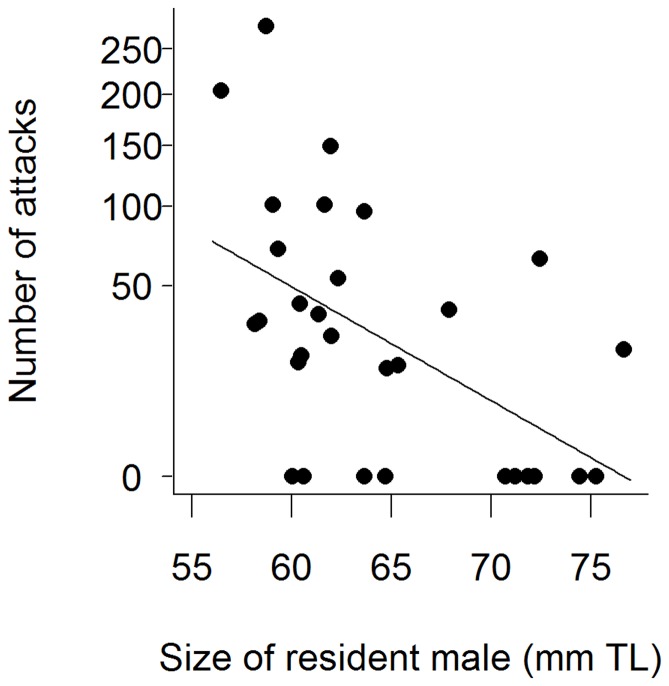
Relationship between the number of attacks (in 30 min) and the size of resident male desert gobies, *Chlamydogobius eremius*. Note square root scale on y-axis.

In the “experimental treatment”, we manipulated perceived mating opportunity by allowing nest holding males to interact with two gravid females prior to the trials. Specifically, two visibly round females (mean total length, TL±SE: 52.5±0.67 mm) were introduced in the smaller of the two compartments (Figure 1). In the “control treatment”, this compartment was left empty. There was no difference in resident male size between the two treatments (TL: 64.5±1.07 mm, two-sample t-test, *t*_28_ = 0.04, *P* = 0.97). After 10 min acclimation, the opaque sheets were lifted, exposing the females (or empty compartment) to the resident but precluding contact, and the tanks were left for 48 h. Presenting females in this way quickly entices resident males to commence courtship [19], [20], and observations confirmed that resident males were indeed courting the females. After 48 h, the opaque sheets were put back, and all females were removed. One male in breeding coloration (hereafter referred to as “intruder males”) was introduced to the front compartment of each tank. Each resident male was given a different intruder male. There was no difference in intruder male size between the two treatments (TL: 59.0±0.99 mm, two-sample *t*-test, *t*_28_ = 1.27, *P* = 0.28). After 10 min acclimation, the opaque sheets were lifted again. The behaviour of the resident males was recorded for 2 hours using a video surveillance system (Signet digital video recorder and cameras, Jaycar, Australia). At the end of the 2 hours, the experiment was terminated and all males were photographed on a 1 cm grid for measurement of total length.

### Video Analysis of Behaviours

The video clips were analyzed (blind with respect to treatment) on a PC which allowed accurate measurements of the different behaviours. The following response variables were quantified: (1) the time from the start of the trial until the first attack (maximum time: 2 h), (2) the intensity of the first aggressive bout (attacks ⋅ min^−1^) within the first 30 min and (3) the total number of attacks within the first 30 min. An “attack” was defined as the resident male darting toward the transparent divider, with fins fully raised and the body unambiguously oriented toward the intruder. “Aggressive bouts” were defined as a series of rapidly repeated attacks ending either with the resident male returning to its nest or being inactive for more than 20 s. Note that we used the intruder males merely as stimuli to elicit an aggressive response in the resident males, and that the main focus was on the initial attack of the resident. This was done to avoid the issues of stimulus habituation, physical exhaustion and the lack of an option to flee that arise when males are in a confined area [11].

### Statistics

All statistical analyses were conducted using the software R 2.10.1 [22]. The time until the first attack was analyzed as a time-to-event analysis, using a Cox proportional hazards model in the “survival” package, version 2.35–8 [23]. This was done to accommodate censoring in the data because some residents did not attack within the 2 h video recording. When the data included influential outliers, we present both analyses with and without these data points. The intensity of the first attack and the number of attacks were analyzed with linear models, and response variables were square-root transformed if this improved normality of model residuals. In all statistical models, the predictor variables were: resident male size (RMS), intruder male size (IMS) and treatment (T, a factor with two levels: either exposed or not exposed to females prior to the trial). We chose to use RMS and IMS as separate predictors rather than some composite measure of “size difference” (e.g. RMS-IMS or RMS/IMS), because the latter may lead to spurious results [24]. Fully factorial models (with all three factors and all four interactions) were simplified by hierarchical deletion of non-significant terms until a minimal adequate model was obtained, using a deletion criterion of P>0.05 [25]. We also performed Bayesian model averaging on the full models, using the BMA package, version 3.13 [26]. This method circumvents the problems inherent in traditional best-model procedures by instead averaging over several models according to posterior model probability. We used the posterior probability of a variable having a non-zero coefficient in the model (Pr_inc_) as a measure of the influence of that variable on the response. That is, Pr_inc_ is the probability that a variable is an contributing factor. As rules of thumb, values of Pr_inc_ <0.50 show no evidence, values between 0.50 and 0.75 show weak evidence and values >0.75 show positive evidence that the predictor is a contributing factor [27].

### Ethics Statement

This study complies with all the relevant Federal and State laws of Australia and was carried out under ethics permit no. BSCI/2007/12 from the Biological Sciences Animal Ethics Committee of Monash University. The Monash University Animal Ethics Committee specifically approved this study.

## Results

### Timing of First Attack

The time until the first attack was not significantly affected by treatment (mating opportunity), intruder male size or any of the interactions (Table 1). However, there was a significant positive relationship between resident male size (RMS) and the time until the first attack (Cox proportional hazards regression model, N = 30, *z* = 2.64, *P* = 0.008). That is, smaller residents attacked earlier than larger residents (Figure 2). Similarly, Bayesian model averaging suggested that RMS was the only factor of importance (Pr_inc_ = 0.82; Table 2). There were seven censored data points, that is, seven of 30 males did not attack within the 2 hr video recording. This frequency was not affected by treatment (control treatment: 3, experimental treatment: 4, Fisher exact test P = 1).

### Attack Intensity

There were no significant effects of treatment, intruder male size or any of the interactions on the intensity of the first bout of aggression (Table 1). However, there was a significant negative relationship between resident male size (RMS) and attack intensity (linear model, R^2 = ^0.20, *F*_1,18 = _4.52, *P* = 0.048; Figure 3). That is, smaller residents attacked the intruder with higher intensity. There were two influential outliers in this data (Cook’s *D*>0.5), and the analysis was performed also without these values. However, removing the outliers only strengthened the result (linear model, R^2 = ^0.38, *F*_1,16 = _9.65, *P* = 0.007; Figure 3). Similarly, Bayesian model averaging attributed resident male size the highest probability of inclusion of all the predictor variables (Table 2). However, the value was low (Pr_inc_ = 0.46) and this is not regarded as positive evidence. Removing the two outliers increased the probability of inclusion of RMS (Pr_inc_ = 0.71; Table 2) which can be considered weak evidence for resident male size being an important factor for attack intensity.

### Number of Attacks

There were no significant effects of treatment, intruder male size or any of the interactions on the total number of attacks resident males performed during the observation period (Table 1). However, there was a significant negative relationship between resident male size (RMS) and the total number of attacks (linear model, R^2 = ^0.28, *F*_1,28 = _10.76, *P* = 0.003; Figure 4). That is, smaller males performed a greater number of attacks. Similarly, Bayesian model averaging suggested that RMS was the only factor of importance (Pr_inc_ = 0.82; Table 2).

## Discussion

Nest-holding desert goby males displayed clear aggression toward intruders. However, timing and intensity of aggression was not affected by our attempt to manipulate their perceived mating opportunities. That is, presenting two gravid females for two days prior to the trial did not influence the response of the residents to intruders, even though resident males had actively courted the females presented to them. In comparison, autumn spiders increase territory and nest defence behaviours when given improved access to mates, probably because this increases the perceived value of the resource [13]. However, other studies have, like ours, failed to find effects of mating opportunities on aggression. For example, in cottonwood borers, *Plectrodera scalator*, prior possession of a female did not affect male aggression [28].

The lack of effect from the females in our study could potentially be explained if the overriding importance of a nest is for a purpose other than breeding. In many substrate brooders, nests also function as shelters for the resident male. In gobies, nests work both as breeding substrates and as refuges [29], [30]. This dual purpose of nests can potentially impact on male defence strategies. For instance, if nest sites are rare and very important as shelters, defence strategies may be less sensitive to perceived mating opportunities. Future studies should investigate if manipulating the quality of the nest/shelter will affect resident male aggression.

The size of the intruding male - either absolute size or size relative to the resident - did not affect resident male aggression. This was contrary to the common assumption that animals should assess their opponents before attacking [1]. Taken together, the lack of effects from the treatment (presence of females) and from intruder size, suggest that nest holding desert gobies have set strategies for initiating aggression. The earliest game-theoretical models often assumed fixed strategies for aggressive behaviours [31]. However, these models have been viewed as too simplistic [1], and have largely been replaced by models that allow for conditional strategies, that is, where the participants change their behaviour depending on the opponents’ appearance and/or behaviour [32]. It is interesting that our data suggest that resident male aggression level was decided before the encounter, in accordance with the simpler evolutionary game models.

We found that smaller residents attacked an intruder sooner and more vigorously than larger residents. This is in contrast with, for example, three-spined sticklebacks, *Gasterosteus aculeatus*, where smaller males were more reluctant to attack intruders [33]. However, other studies have found, like us, that smaller males can be more aggressive. For instance, in jumping spiders, *Plexippus paykulli*, smaller individuals are more likely to escalate fights [24]. Likewise, in swordtails, *Xiphophorus* spp., smaller males are more prone to initiate and escalate aggression [6], [34], [35]. Why should small males behave in this way? It is well established that larger body size is an important predictor for winning escalated fights [36], [37], [38]. Indeed, in situations where two opponents are forced to “fight it out”, the smaller, more aggressive, male will typically end up losing [24], [39]. Hence, *prima facie*, it may seem paradoxical for smaller males to have a high aggression strategy. Several evolutionary game models have attempted to explain this apparent paradox [the so called ‘Napoleon complex’, 40,41,42,43]. These models suggest that a high aggression strategy in small males may be adaptive (i.e. evolutionarily stable) if one assumes either assessment error on the part of the smaller male [40], [42], or that there is a some chance of the smaller male actually winning an escalated combat [41]. These models attempt to explain observations that small males initiate fights they are likely to lose. However, because escalation of fights is typically rare in the wild, it may be misleading to use the losses of smaller males in confined laboratory conditions as the premise of whether a high aggression strategy is beneficial or not [11]. We suggest that if intruders are more likely to flee than retaliate in the wild, small residents may in fact benefit from attacking early, before the intruder can assess him or his resource.

Resident males in our experimental set up had access to both visual and olfactory cues. However, other sensory cues may also be important. The lateral line system of gobies, for example, contain superficial and canal neuromasts that are distributed in patterns different from those in other fish families [44]. The potential role for neuromasts in mechanosensory intruder detection [45], especially in absence of visual cues in turbid desert waters, would be an interesting topic for further research.

In conclusion, we found nest-holding male aggression to be independent of perceived mating opportunities and of the size of the intruding male. Instead, residents appeared to have set strategies for initiating aggression related to their own body size. In order to examine the adaptive value of the commonly observed high aggression strategy in small males, future studies should be conducted under field-like conditions that allow the full range of behavioural options for both residents and intruders.
